# Successful endoscopic endonasal trans‐sphenoidal resection for pituitary macro adenoma with apoplexy in a twin pregnancy: A case report

**DOI:** 10.1002/ccr3.9172

**Published:** 2024-07-10

**Authors:** Ujjwal Kumar Jha, Pragyan Basnet, Sunil Kumar Das

**Affiliations:** ^1^ Patan Academy of Health Sciences Lalitpur Nepal; ^2^ General Practice and Emergency Medicine Rampur Hospital Rampur Palpa Nepal

**Keywords:** emergency medicine, endocrinology and metabolic disorders, fetal medicine, obstetrics/gynecology, perinatal medicine

## Abstract

**Key Clinical Message:**

Pituitary apoplexy is a medical and surgical emergency requiring prompt diagnosis and often urgent treatment to manage symptoms and prevent further complications.

**Abstract:**

This report describes the successful management of a 37‐year‐old pregnant woman with a history of pituitary macroadenoma and apoplexy during a twin pregnancy. Presenting with bitemporal vision loss, a common pituitary adenoma symptom, she showed no other alarming signs despite a twin pregnancy. Successful endoscopic resection improved her vision, and postoperative recovery was uneventful. The discussion underscores significance of the diagnostic utility of contrast MRI. The patient's favorable outcome supports endoscopic resection feasibility in pregnant individuals with pituitary apoplexy.

## 1 INTRODUCTION

Pituitary adenomas are benign tumors arising from anterior pituitary.^1^ Pituitary adenoma can be classified based on hormone production (functional and non functional) and size (microadenoma [<10 mm] and macroadenoma [>10 mm]).^2^, ^3^ Both functional and non‐functional adenomas can produce various symptoms (most commonly headache) due to mass effect and compression of surrounding structures.^4^

Pituitary apoplexy, a rare condition involving hemorrhage or infarction in the pituitary gland, primarily associated with adenomas, may result from insufficient blood supply in large tumors, increased blood vessel fragility, or compression, with risk factors including surgery, head trauma, anticoagulation, and pregnancy.^5^ Pituitary apoplexy occurring in pregnancy is an exceedingly rare occurrence, and if not identified, it poses a potentially life‐threatening risk to both the mother and the fetus.^6^ Severe sudden onset headache is the typical clinical presentation of the patient which may be associated with visual changes.^4^

## 2 CASE HISTORY

A 37‐year‐old Gravida 4 Para 1 Living 1 Abortion 2, at 25 weeks of gestation with twin pregnancy presented to the outpatient department with a history of decreased vision at the peripheral area for a year, which had worsened over the last 6 months. She had a history of pituitary macroadenoma and had planned IVF pregnancy, which turned out to be diamniotic dichorionic twin pregnancy. There was no history of fever, headache, loss of consciousness, abnormal body movements, nausea and vomiting. On examination, her general condition was conscious, oriented with Glassgow Coma Scale 15/15. Her vision was impaired, and perimetry showed bilateral temporal hemianopia. Despite known case of pituitary macro adenoma other differential diagnosis of decreased vision during pregnancy like gestational diabetes, pre eclampsia, eclampsia, migraine, and retinal detachement was also considered. Patients sugar profile was within normal limit, blood pressure within normal range, no evidence of proteinuria, retinoscopy revealed normal retina, likewise clinical examination and history excluded migraine.

## 3 METHODS

Gadolinium contrast MRI of the brain and orbit showed well defined high T2/FLAIR and iso T1 signal intensity lesion in sella and supra sella region causing mild widening of sella with T2 low signal intensity area in dependent part showing SWI blooming, mild compressing optic chiasma and just abutting cavernous part of left ICA Differential diagnosis, macroadenoma with apoplexy (Knosp grade 1 in left side).

Her prolactin level was 124.44 ng/mL which was normal (normal range for prolactin during pregnancy 80–400 μg/L) for the pregnant population, however it is about 4 times higher than the non‐pregnant group. Growth hormone, serum cortisol, thyroid stimulating hormone and anti‐mullerian hormone were within normal limits pre operatively.

The patient underwent endoscopic endonasal trans‐sphenoidal resection of the pituitary adenoma. She was extubated on the same day and was shifted to the Neurosurgical intensive care unit (NSICU). She was consulted by the gynecological and ENT team. Her nasal pack was removed on post‐operative day (POD) 3, and her ICU stay was uneventful. Her hormonal profile was within normal limit on 48 h and after 7 days post operatively. She was shifted to the ward on POD‐5, and visual field tests showed improved peripheral vision on subsequent days.

The patient was discharged on post‐operative day 7 and advised to follow‐up after 1 week in the neurosurgery outpatient department with perimetry test. Her Glasgow Coma Scale (GCS) was 15/15, and she had no focal neurological deficits. The baby's fetal heart sounds were within normal limits, and fetal movements were felt by her. All danger signs were explained, and the patient was discharged with advice to follow‐up on antenatal care.

## 4 CONCLUSION AND RESULT

Impaired peripheral vision, accompanied by headaches or other endocrinological symptoms, can indicate a possible pituitary adenoma and its apoplexy. While elevated prolactin levels are normal during pregnancy, they may also point to a prolactin‐secreting adenoma. MRI is a safe diagnostic method for detecting pituitary apoplexy in pregnant women. When surgery is needed, Endoscopic Endonasal Trans‐Sphenoidal Resection effectively improves visual symptoms and palsy.

Patient delivered male infant of 2300 g (APGAR 7/10, 9/10) and female infant of 2450 g (APGAR 7/10 9/10) via elective lower section cesarean section.

Patient was advised for regular out patient department follow up for hormonal studies, visual evaluation and repeat MRI after 1 year of the surgery.

## 5 DISCUSSION

Pituitary macroadenoma should be differentiated from oligodendroglioma, meningioma, hemangioblastoma, astrocytoma, schwannoma, primary CNS lymphoma, medulloblastoma, ependymoma, craniopharyngioma, pinealoma, AV malformation, brain aneurysm which can be diagnosed by neuroimaging and hormonal profile. Aforementioned differential can be diagnosed by neuro imaging and hormonal studies. The mass effect of pituitary adenoma, affecting approximately 40%–60% of patients, is primarily attributed to the suprasellar extension of the tumor, leading to visual changes, with bitemporal hemianopia being the most prevalent type.^1^ During pregnancy, elevated prolactin hormone levels are typical, and while the prolactin hormone level is high in this case, it falls within the normal range for pregnant women, suggesting that the increase may be attributed to pregnancy alone or in conjunction with a prolactin‐secreting pituitary adenoma.^6^ Similarly other hormones secreted by anterior pituitary like GH, ACTH, and TSH should also be investigated.^1^

MRI is a safe diagnostic tool throughout any trimester of pregnancy, detecting up to 90% of pituitary apoplexy, where it is visualized as a suprasellar mass on T1 weighted scans, with hemorrhage appearing hyperintense compared to the rest of the brain; additionally, MR angiogram aids in distinguishing pituitary apoplexy from an aneurysm.^6^

While pituitary apoplexy was historically deemed a neurosurgical emergency, contemporary trends indicate a shift towards conservative approaches among surgeons, with instances of spontaneous resolution and tumor disappearance gaining recognition in recent years.^7^

Endoscopic Endonasal Trans‐Sphenoidal Resection, as a minimally invasive approach, offers effective treatment for patients with pituitary apoplexy at various stages of admission, providing rapid decompression and facilitating swift patient recovery; post‐surgery, there is approximately 90% improvement in visual symptoms and around 80% improvement in visual palsy.^8^ Nearly 80% of patients will require hormone replacement after pituitary apoplexy, including corticosteroids (40%–85%), thyroid hormone (50%–70%), desmopressin (6%–25%), sex steroids (40%–80%), and with growth hormone deficiency reported in 16% of cases.^9^

## AUTHOR CONTRIBUTIONS

**Sunil Kumar Das:** Writing – original draft; writing – review and editing. **Ujjwal Kumar Jha:** Writing – review and editing. **Pragyan Basnet:** Supervision.

## FUNDING INFORMATION

None.

## CONFLICT OF INTEREST STATEMENT

The authors declare no conflicts of interest.

## CONSENT

Written informed consent was obtained from the parents to publish this report in accordance with the journal's patient consent policy.

## Data Availability

Data will be provided by the corresponding author upon reasonable request.
